# The public health exposome and pregnancy-related mortality in the United States: a high-dimensional computational analysis

**DOI:** 10.1186/s12889-022-14397-x

**Published:** 2022-11-17

**Authors:** E. W. Harville, S.K. Grady, MA Langston, P. J. Juarez, D. Vilda, M. E. Wallace

**Affiliations:** 1 Department of Epidemiology, Tulane University School of Public Health and Tropical Medicine, New Orleans, LA USA; 2 Department of Electrical Engineering and Computer Science, University of Tennessee, Knoxville, TN USA; 3 Department of Family and Community Medicine, Meharry Medical College, Nashville, TN USA; 4 Department of Social, Behavioral, and Population Sciences, School of Public Health and Tropical Medicine, Tulane University, New Orleans, LA USA

**Keywords:** Maternal mortality, Exposome, Ecological, Disparities, Public health

## Abstract

**Background:**

Racial inequities in maternal mortality in the U.S. continue to be stark.

**Methods:**

The 2015–2018, 4-year total population, county-level, pregnancy-related mortality ratio (PRM; deaths per 100,000 live births; National Center for Health Statistics (NCHS), restricted use mortality file) was linked with the Public Health Exposome (PHE). Using data reduction techniques, 1591 variables were extracted from over 62,000 variables for use in this analysis, providing information on the relationships between PRM and the social, health and health care, natural, and built environments. Graph theoretical algorithms and Bayesian analysis were applied to PHE/PRM linked data to identify latent networks.

**Results:**

PHE variables most strongly correlated with total population PRM were years of potential life lost and overall life expectancy. Population-level indicators of PRM were overall poverty, smoking, lack of exercise, heat, and lack of adequate access to food.

**Conclusions:**

In this high-dimensional analysis, overall life expectancy, poverty indicators, and health behaviors were found to be the strongest predictors of pregnancy-related mortality. This provides strong evidence that maternal death is part of a broader constellation of both similar and unique health behaviors, social determinants and environmental exposures as other causes of death.

**Supplementary Information:**

The online version contains supplementary material available at 10.1186/s12889-022-14397-x.

## Synopsis

Study Question: What can analyzing the exposome tell us about maternal mortality?

What’s already known: Several individual-level medical and social factors raise the risk for maternal mortality. Analyzing a large number of predictors simultaneously is computationally difficult

What this study adds: A high-dimensional analysis found county-level overall life expectancy, poverty indicators, and health behaviors to be the strongest predictors of pregnancy-related mortality when an nationwide analysis of the public health exposome was conducted.

## Introduction

Maternal mortality (MM) has become a topic of intense interest in recent years. Statistics suggesting an increase in maternal mortality in the U.S. at a time when most of the world was seeing a decline [1], as well as the stark racial divides [2], has created renewed interest. In 2020, the National Center for Health Statistics (NCHS) reported a national MM ratio (MMR, defined as deaths of women while pregnant or within 42 days of being pregnant, from any cause related to or aggravated by the pregnancy or its management (but not from accidental or incidental causes) at 17.4 per 100,000 live births for 2018, up from to 12.7 in 2007 [3], an increase of 37%. Similarly, the pregnancy-related mortality ratio (PRM, defined as maternal death during pregnancy or within 1 year from any cause related to or aggravated by the pregnancy or its management) has increased 238%, from 7.2 deaths per 100,000 live births in 1987 to 17.2 deaths per 100,000 live births in 2011–2015 [4]. Almost a third of pregnancy-related deaths occur during pregnancy, approximately half of all occur after the day of delivery, and the remainder occur on the day of delivery [5, 6]. Additionally, wide racial inequities in maternal death have been found, with non-Hispanic (NH) Black women being two to four times more likely to die from a pregnancy-related complication than NH White women [3, 4, 6].

While individuals’ biological, behavioral, and demographic characteristics occur within PRM and are inseparable from the social and economic context of the environments in which they live, efforts to understand maternal death are just beginning to extend beyond consideration of individual-level risk factors. The literature on place-based/contextual determinants of maternal mortality remains very sparse, especially in comparison to other reproductive health outcomes, largely due to the rarity of this outcome and unavailability of administrative data with geographic identifiers to achieve adequate sample sizes. Research on this outcome has had a historically narrow focus on clinical factors and medical interventions; a recent systematic review of social determinants of maternal morbidity and mortality concluded that “relatively little attention has been given to examining social determinants of health and maternal health outcomes in the US”, and identified only 5 studies from 1990 through 2018 that investigated associations between “area-level characteristics” and maternal mortality [7]. Ecological systems theory suggests that MM is affected by a range of factors at the societal, policy, community, interpersonal, and individual levels [8]. A recent analysis of county- and zip code-level predictors found that social factors such as income, education, and food access were most strongly associated with pregnancy-related mortality [9]. Most risk factors in that analysis were similar between Black and White PRM but the absolute effect was often stronger for the Black PRM.

**Fig. 1:**
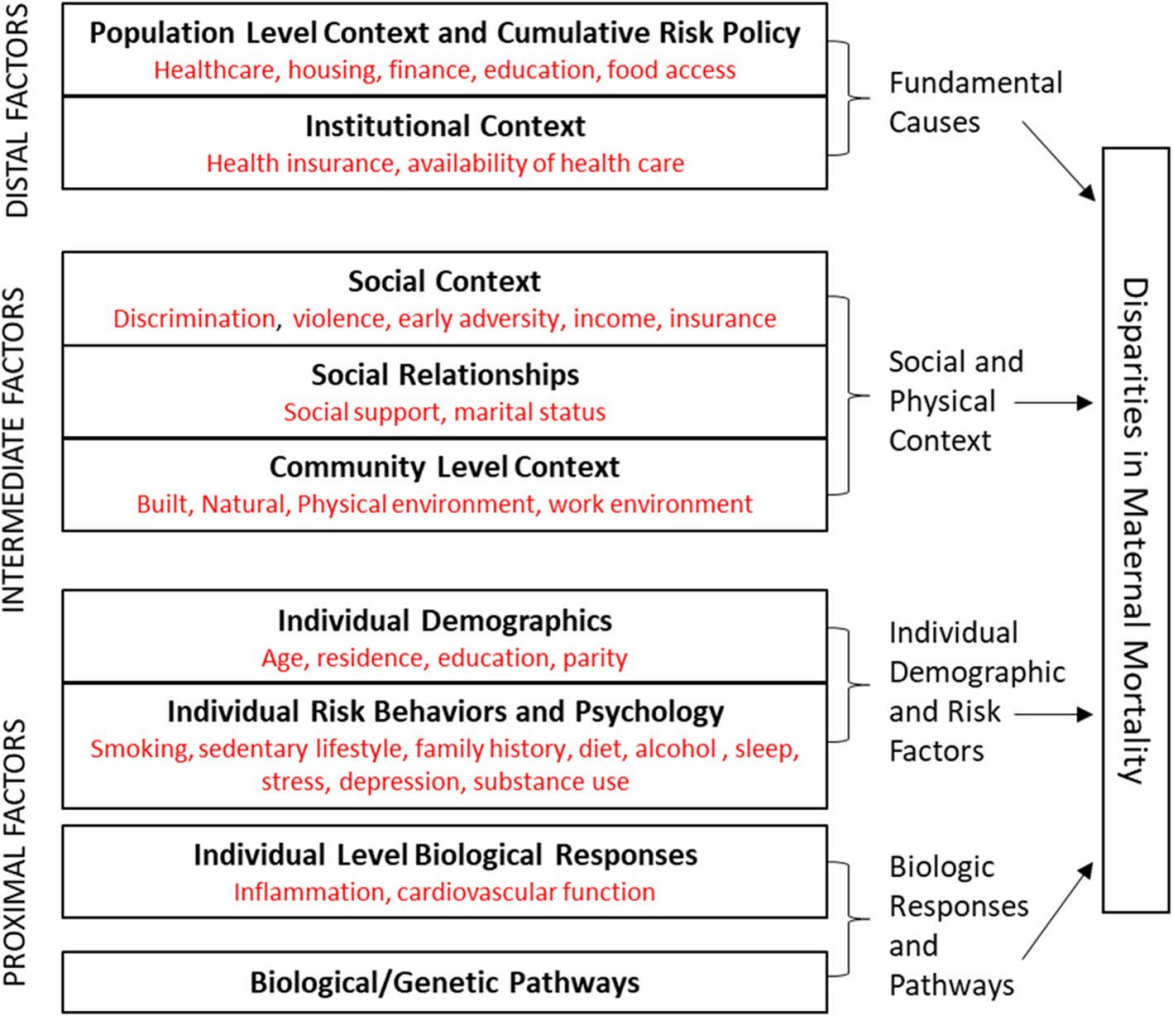
Conceptual model for disparities in maternal mortality

Disentangling the web of causation underlying MM and MM disparities is a challenging problem, but may be better understood with an exposome lens. An exposome approach considers how cumulative and lifetime environmental exposures across different domains are related to MM (Fig. 1). The “public health exposome” (PHE) in particular allows for the simultaneous evaluation of individual characteristics and behaviors within the context of exposures from a number of different domains, including the natural, built, social and policy environments, and health and health care. Social determinants such as racial residential segregation in US communities and other features of structural racism in US governance, institutions, cultural norms and values [10], have been identified as fundamental causes of racial health inequities, including maternal health [11, 12]. While the built environment has not been directly studied with respect to maternal mortality, a number of factors have been found to affect pregnancy health [13–16], including lack of transportation, access/availability of healthy foods, access to quality health care, and safe physical activity spaces, undergirded by social determinants such as high rates of poverty and low educational attainment within communities. Exposure to chemicals in the natural environment, especially air pollutants such as particulate matter and nitrogen oxides, previously have been associated with maternal mortality in studies conducted in Asia and Africa [17, 18]. Thus, a model that allows for the simultaneous consideration of complex, real world exposures from the social, healthcare, built, natural, and policy environments and PRM is warranted.

While incidence of PRM is unacceptably high, maternal deaths are rare in absolute terms. Many state-wide reviews include less than 100 cases [19–24]. Analyzing a concept like the exposome – the totality of exposures – even at the ecological level requires methods that can handle thousands of exposures simultaneously. When combined with a small number of cases and the totality of interconnected, multi-level exposures, contexts, and pathways occurring across the life course that shape a person’s risk for death during reproduction, the analytic problem becomes particularly complicated. Complex computational methods provide an alternative methodology for examining large datasets, patterns and places of occurrence, and contributing factors to PRM. A main aim of this study was to apply a high-dimensional exposome-wide analysis to pinpoint novel patterns of associations of risk and protective factors of PRM across counties in the United States in an effort to identify and elucidate associations across the natural built, social and policy environments, health and healthcare. This analysis uses novel methods of data reduction, graph-theoretical algorithms, and Bayesian analysis to examine an extensive dataset with a wide conceptual and geographical range.

## Methods

### Outcome data

**Fig. 2:**
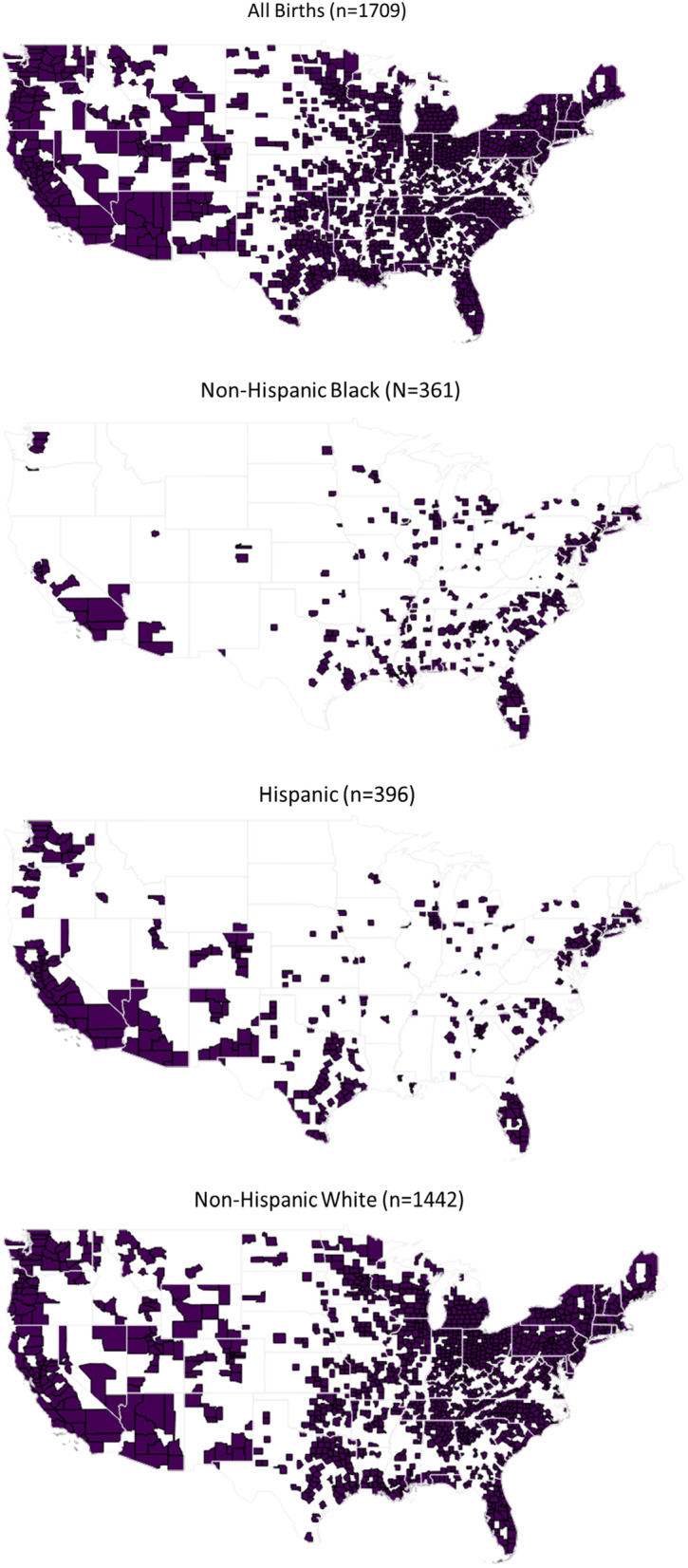
Counties included in the analysis (*N* = 1709)

The primary outcome in this analysis was the county-level pregnancy-related mortality ratio (PRM; deaths per 100,000 live births). (The analysis uses county equivalents in states that have different administrative units; for brevity, “county” is used in this paper as a generic term.) All cases of pregnancy-related death were identified in the 2015–2018, restricted use, maternal mortality file obtained through the NCHS using International Classification of Disease-10^th^ revision codes for underlying cause of death (A34, and all codes in the Pregnancy, Childbirth, and the Puerperium Classification [O00-O99]). The restricted use maternal mortality file includes death records with geographic identifiers for county of residence for every decedent in the United States and applies the 2018 vital records coding scheme for identifying maternal deaths. The revised coding scheme mitigates misclassification errors resulting from the adoption of a standardized pregnancy-status checkbox on revised death certificates [3]. To maximize rate stability, counts of deaths were summed over four years in each county where a minimum of 1000 live births occurred in the same time period (*n* = 1709; without this exclusion, a few counties with a low number of births had artifactually high rates; Table S1); no exclusions were made with respect to the number of maternal deaths (Fig. 2). Compared to the included counties, excluded counties with low numbers of births (Table S1) had smaller populations; somewhat lower median income (45,400 vs. 51,300 USD); and a lower percentage NH Black population (median 0.9% vs. 4.2%), consistent with rural counties largely located in the Midwest and West (Fig. 2). Counts of live births by county were aggregated from the NCHS restricted use natality files and summed from 2015–2018 in order to estimate county-level PRM as deaths per 100,000 live births.

### The PHE

A large data repository [25] has been collected, collated and curated, providing information on the natural, built, social and policy environments and health and health care, incorporating data from local, national, and global sources (Table 1). Variables were geocoded at scales ranging from 1-km grid cell, to census tract, and county, areas, linked by cross-walks. The PHE data repository contains measures of both health and environmental exposures for 3,141 counties and county equivalents, and spans over 15 years (2003–2018). It currently stores over 62,000 variables that have been geocoded and harmonized at an annual and county level in order to provide the spatial–temporal, contextual environmental data that can be linked to residential addresses and used to analyze environmental context at the county level. The PHE is fully curated with meta-data and a searchable data dictionary. These data were restricted to 2014–2018 in order to coincide with the time frame of the PRM data used in this study.

**Table 1:** Public health exposome database

Domain	Definition	Sample Data Sources
Built Environment	physical characteristics of places we live, work and play; patterns and types of development; building location and design, and transportation infrastructure	North American Land Data Assimilation System, US Department of Agriculture (Food Environment Atlas), US Department of Interior (National Land Cover/Land Use), US Post Office (residential and business vacancies)
Social Environment	social/cultural norms, economic employment policies and systems, the distribution of money, power, and resources, and political systems	Department of Labor (unemployment statistics) Department of Education (graduation rate, student enrollment, adult literacy), Agency for Toxic Substances and Disease Registry (Social Vulnerability Index), US Census, American Community Survey
Natural Environment	climate, weather, and natural resources that affect human survival and economic activity; chemical exposures found in the air, water, and land	National Oceanic and Atmospheric Administration, Federal Emergency Management Agency (national flood hazard layer) US Environmental Protection Agency (EJ Screen, Superfund sites, Environmental Quality Index, National Air Toxics Assessment), US Geological Survey (water usage), PM2.5, temperature, heat index
Health	health care availability, health outcomes	CDC Wonder, Dartmouth Atlas (Medicare claims data), AIDSVU, HRSA Data Warehouse (health professions shortage areas, medically underserved areas), Health Indicators Warehouse (morbidity, mortality), RWJ Foundation (County Health Rankings)
Policy	government expenditures, benefit programs	Veterans’ compensation, Federal government expenditures, employment, and procurement; Medicare; Sales taxes; local government finances

### Computational analysis

**Fig. 3:**
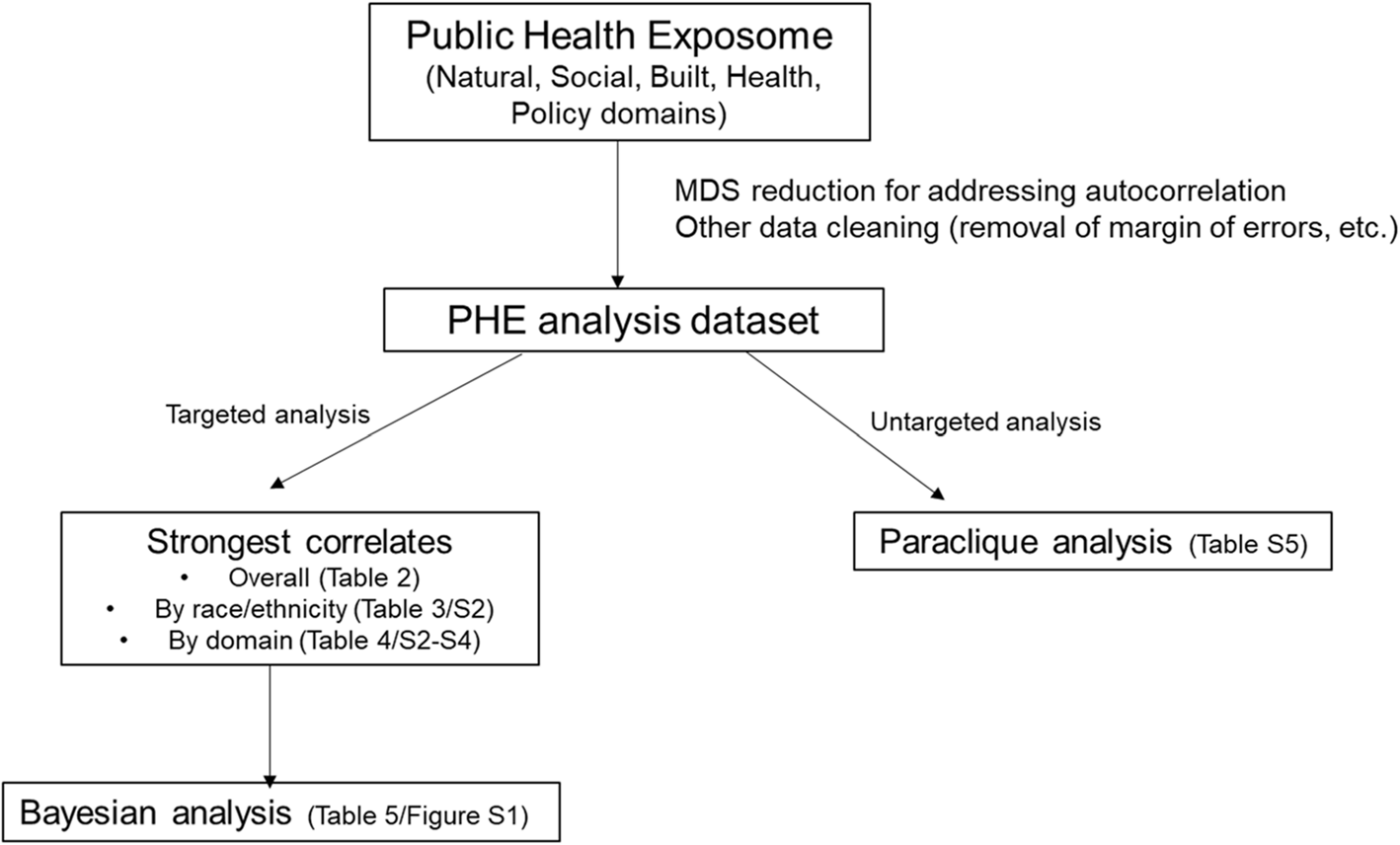
Overview of analysis structure

An overview of the analysis flow is provided in Fig. 3. Idiosyncrasies of information capture and variable overlap tend to make data such as this rife with repeated and collinear measures, which can clog the analysis and obfuscate potential results. An exposome autocorrelation guideline [26] was therefore adopted by which variables may be assumed to provide the same information whenever their correlation is at least 0.9. Pearson’s coefficient was chosen because all variables passed the Wilk-Shapiro [27] normality test after correcting for multiple tests using the Benjamini and Hochberg false discovery rate method [28]. Graph theoretical screening algorithms were then applied as follows. A finite, simple, undirected graph was constructed with a vertex for every variable and with edges only between autocorrelates as just defined. Integer Linear Programming via the Python MIP Optimizer [29] was applied using default software settings to compute a minimum dominating set [30]. Vertices not in this set were removed, reducing the focus to 3,903 variables. Confidence limit and margin of error variables were also eliminated, leaving 1591 variables for subsequent analysis.

County-level total population, NH White, NH Black, and Hispanic PRM ratio variables were then linked to the PHE by Federal Information Processing System (FIPS) codes for state and county. To this dataset, scalable computational methods [31] were applied in both a supervised, targeted fashion and an unsupervised, data science approach.

Supervised analysis was focused exclusively on the four PRM ratio variables. Features for each of these variables were first restricted to county equivalents (counties, parishes, and boroughs) with 1000 or more live births for the respective demographic. This limited the analysis to 1709 county equivalents for total population, 1442 for NH White, 361 for NH Black, and 396 for Hispanic population (Fig. 2). These four feature sets were then used to compute four sorted lists of Pearson correlation coefficients, one list for each PRM ratio variable and all its PHE correlates. Each list was then subdivided into five sublists based on PHE domains of interest (natural, built, social, policy, and health). The result was 24 lists (five domain lists and one combined list for each of the four PRM ratio variables). Correlates with *r* > 0.15 from each list were then retained for detailed scrutiny. Domains are defined by data source. Thus, for example, the built domain may include variables about income as it relates to housing programs, while the health and health care domain may include variables from morbidity, mortality, and health behavior and health access data.

Unsupervised analysis was more complex and performed in an effort to discover unseen variable networks or relationships in which PRM may play a role. Paraclique analysis is resilient to noise, has high density guarantees, and is robust [32] across multiple parameter settings. It began with the construction of a complete, finite, simple, undirected graph whose vertices denoted PHE and PRM variables and whose edge weights recorded pairwise Pearson correlation coefficients computed over the 1709 county equivalents with 1000 or more total population live births. Completeness and edge weights were eliminated by thresholding. Data inhomogeneity ruled out the use of a single threshold, as is commonly done for example with spectral methods [33], and so absolute values of the correlation coefficient of 0.15, 0.2, 0.3, 0.5, 0.7, and 0.8 were chosen for study. Dense subgraphs in the form of paracliques [34] were then extracted using glom terms [35] ranging from one to three for each of these five graphs A paraclique was retained for further analysis whenever it contained at least one PRM ratio variable.

Both supervised and unsupervised analyses produced variable sets of manageable size, to which traditional but unscalable methods were then applied. Bayesian network analysis was performed using the *bnlearn* R package [36], with a greedy hill-climbing method employed, again with default settings, to search for a network with minimal Bayesian information criterion score. A sample Bayesian network for the total population PRM ratio variable and its top 20 correlates from each of the PHE domains is illustrated with the finite, simple, directed graph shown in Figure S1.

## Results

When total population PRM was considered across the PHE in the supervised analysis, the variable most strongly correlated was years of potential life lost (Pearson correlation coefficient *r* = 0.254), followed by an inverse correlation with overall life expectancy (*r* = -0.249). Other correlated indicators were smoking, overall poverty, heat, lack of exercise, and lack of adequate access to food (Table 2). When stratified by race/ethnicity, several similar factors emerged (Table 3). For PRM in the NH Black population, the most highly correlated variables were publicly supplied per capita water use (*r* = 0.206); an inverse association with births to unmarried, White NH women (*r* = -0.206); households headed by younger people (*r* = 0.194); lack of sleep (*r* = 0.192); poor physical health (*r* = 0.184); and mental health (0.164); and several indicators of poverty, heat, and sun exposure. The publicly supplied per capita water use correlation seemed to be due to a single parish in Louisiana, which had notably high water use and NH Black PRM; if eliminated from the analysis, the correlation was much lower (*r* < 0.15). PRM in the Hispanic population was most strongly correlated with lower income in households living in public housing (*r* = 0.167) birth rates to those aged 15–17 (*r* = 0.167), and proportion of residents with a high school degree (*r* = -0.157). The NH White population PRM was associated with overall life expectancy (*r* = -0.188), household income and poverty (*r* = 0.181), lack of physical activity (*r* = 0.180), lack of health insurance (*r* = 0.176), and population with less than a high school degree (*r* = 0.174).

**Table 2:** Factors most strongly associated with national 4-year total population pregnancy-related mortality ratio per 100,000 live births (2015–2018). Correlations are with respect to the relevant PRM ratio variable

Variable Description	Correlation
Years of potential life lost before age 75 per 100,000 population (age-adjusted)	0.254
Average number of years a person can expect to live	-0.249
Percentage of adults who are current smokers	0.246
Percent of population living in poverty, 2016	0.241
Percentage of adults aged 20 and over reporting no leisure-time physical activity	0.237
Percentage of population who lack adequate access to food	0.232
Percent Persons in Poverty, 2015	0.231
Sum of series for Socioeconomic theme^a^	0.229
Index of factors that contribute to a healthy food environment, from 0 (worst) to 10 (best)	-0.228
Births unmarried non-Hispanic women	0.220
Median Household Income, 2016	-0.219
Percentage of adults reporting 14 or more days of poor physical health per month	0.211
Birth Rates, ages 15–19, 2014	0.201
Percentile Percentage of persons with no high school diploma(age 25 +) estimate	0.200
Sum of series for Household Composition theme^b^	0.196
Percentage of children that live in a household headed by single parent	0.193
Number of extreme heat days: Heat Index, 105°F	0.192
Percent Living in Poverty, 2015	0.192
Percentage of adults who report fewer than 7 h of sleep on average	0.192
Birth Rates, 2018–2019, 2014	0.190
Percentage of population that is non-Hispanic Black or African American	0.189
Percentile percentage of civilian noninstitutionalized population with a disability estimate	0.187
Average gross household contribution towards rent per month (includes payment toward rent and utilities) (Excludes zero values and missing values) for Program 3 = Housing Choice Vouchers, 2017	-0.186
Percentage of households that have income in the bracket of $20,000 or more yearly for Program 5 = Project Based Sect. 8 = 202/PRAC, 2018	-0.185
Average number of mentally unhealthy days reported in past 30 days (age-adjusted)	0.185
Percentage of adults aged 20 and above with diagnosed diabetes	0.184
Percentage of adults ages 25–44 with some post-secondary education	-0.184
Free school lunch eligibility (percent)	0.184
Per Capita Personal Income, 2016	-0.182
Average gross household contribution towards rent per month (includes payment toward rent and utilities) (Excludes zero values and missing values) for Program 5 = Project Based Sect. 8 = 202/PRAC, 2017	-0.181
Median Household Income dollar, 2015	-0.180
Percentage of households that have income in the bracket of $20,000 or more yearly for Program 5 = Project Based Sect. 8 = 202/PRAC, 2017	-0.179
Percentage of the adult population (age 20 and older) that reports a body mass index (BMI) greater than or equal to 30 kg/m2	0.179
Percentage of households that have income in the bracket of $5000-$9999 yearly for Program 2 = Public Housing, 2017	0.179
National School Lunch Program participants (% pop), 2014	0.178
Percent of population without health insurance	0.178
Percentage of households that have income in the bracket of $5000-$9999 yearly for Program 1 = Summary of All HUD Programs, 2018	0.177
Percentage of children enrolled in public schools that are eligible for free or reduced price lunch	0.176
% of the population below poverty level in the census tract where HUD assisted families reside (Census 2010 designation) for Program 3 = Housing Choice Vouchers, 2018	0.175
Percentage of households that have income in the bracket of $5000-$9999 yearly for Program 2 = Public Housing, 2015	0.175
Number of deaths due to firearms per 100,000 population	0.174
Percentage of households that have income in the bracket of $20,000 or more yearly for Program 5 = Project Based Sect. 8 = 202/PRAC, 2016	-0.170
Number of extreme heat days: Heat Index, 95°F	0.169
Percentage of households that have income in the bracket of $20,000 or more yearly for Program 5 = Project Based Sect. 8 = 202/PRAC, 2014	-0.168
Percentage of adults reporting binge or heavy drinking	-0.168
Percentage of households that have income in the bracket of $5000-$9999 yearly for Program 1 = Summary of All HUD Programs, 2015	0.167
School Breakfast Program participants (% pop), 2014^a^	0.166
Percentage of households that have income in the bracket of $5000-$9999 yearly for Program 2 = Public Housing, 2016	0.166
Percentage of households in which the race of the head of household is Black and the ethnicity is not Hispanic for Program 1 = Summary of All HUD Programs, 2018	0.165
Number of extreme heat events: Heat Index, 95°F, 3 days minimum	0.164
Average gross household contribution towards rent per month (includes payment toward rent and utilities) (Excludes zero values and missing values) for Program 2 = Public Housing, 2014	-0.164
Rate of HIV infection among MSM, 2018	0.163
Percent with a High School Education, 2014	-0.163
Percentages of households headed by female for Program 3 = Housing Choice Vouchers, 2015	0.161
Age-adjusted rate of death from Heart Attack among persons 35 and older per 100,000 population	0.160
Ratio of household income at the 80th percentile to income at the 20th percentile	0.159
Average total household income per year for Program 3 = Housing Choice Vouchers, 2016	-0.159
Percentage of households that have income in the bracket of $5000-$9999 yearly for Program 2 = Public Housing, 2014	0.159
Percentage of households in which the older of the household head or spouse is 24 or less years of age for Program 1 = Summary of All HUD Programs, 2016	0.158
Age-adjusted rate of death from Heart Attack among persons 35 and older per 100,000 population	0.158
Birth Rates among white women aged 40–54	-0.157
Percentage of households that have income in the bracket of $20,000 or more yearly for Program 5 = Project Based Sect. 8 = 202/PRAC, 2015	-0.157
Percentage of households that have income in the bracket of $5000-$9999 yearly for Program 3 = Housing Choice Vouchers, 2016	0.156
Percentage of households that have income in the bracket of $20,000 or more yearly for Program 1 = Summary of All HUD Programs, 2017	-0.156
Average gross household contribution towards rent per month (includes payment toward rent and utilities) (Excludes zero values and missing values) for Program 3 = Housing Choice Vouchers, 2015	-0.156
Number of extreme heat events: Heat Index, 100°F, 3 days minimum	0.156
Standardized Per Capita Medicare Cost, 2014	0.155
Average gross household contribution towards rent per month (includes payment toward rent and utilities) (Excludes zero values and missing values) for Program 2 = Public Housing, 2017	-0.155
Percent of mobile-home housing units	0.155
Number of newly diagnosed chlamydia cases per 100,000 population	0.154
Percentile percentage of single parent households with children under 18	0.153
Percentage of households that have income which comes in the bracket of $1-$4999 yearly for Program 5 = Project Based Sect. 8 = 202/PRAC, 2018	0.153
Flag—the percentage of single parent households is in the 90th percentile (1 = yes,0 = no)	0.153
Average gross household contribution towards rent per month (includes payment toward rent and utilities) (Excludes zero values and missing values) for Program 2 = Public Housing, 2016	-0.152
Percentage of households that have income in the bracket of $20,000 or more yearly for Program 3 = Housing Choice Vouchers, 2018	-0.152
Percentage of population with adequate access to locations for physical activity	-0.151
Preventable Hospital Stays Rate, 2015	0.151
Average total household income per year for Program 1 = Summary of All HUD Programs, 2017	-0.151
Average gross household contribution towards rent per month (includes payment toward rent and utilities) (Excludes zero values and missing values) for Program 2 = Public Housing, 2015	-0.150
Rate of hospital stays for ambulatory-care sensitive conditions per 100,000 Medicare enrollees	0.150
Percentages of households headed by female for Program 1 = Summary of All HUD Programs, 2016	0.150

*HUD* Housing and Urban Development, *PRAC* Project Rental Assistance Contract, *MSM* men who have sex with men

^s^socioeconomic theme: below poverty, unemployed, income, no high school diploma

^b^household composition theme: aged 65 + , aged < 18, civilian with a disability, single-parent household

**Table 3:** Factors from all PHE domains most strongly associated with national 4-year pregnancy-related mortality ratio per 100,000 live births (2015–2018), stratified by race/ethnicity. Correlations are with respect to the relevant PRM ratio variable

Maternal race/ethnicity	PHE variable description	Correlation
Non-Hispanic Black		
	Domestic, publicly supplied per capita use, in gallons/day [DO-PSDel/PS-TOPop], 2015	0.206
	Births to unmarried women, white non-Hispanic, 2014	-0.206
	Percentage of households in which the older of the household head or spouse is 24 or less years of age for Program 3 = Housing Choice Vouchers, 2014	0.194
	Percentage of adults who report fewer than 7 h of sleep on average	0.192
	Average household income as a percent of local area median family income as defined by HUD, and adjusted for household size for Program 2 = Public Housing, 2014	0.184
	Daily county-level population weighted spectral irradiance at local solar noon time at 305 nm. (Jul), 2015	0.180
	Percentage of households in which the older of the household head or spouse is 24 or less years of age for Program 3 = Housing Choice Vouchers, 2015	0.180
	Percentage of adults reporting 14 or more days of poor physical health per month	0.177
	Number of extreme heat days: Maximum Temperature, 90°F	0.174
	Number of extreme heat days: Heat Index, 105°F, 2014	0.170
	Sum of series for Socioeconomic theme, 2014^a^	0.169
	% of households who were in the program less than a year from the date of Picture snapshot for Program 3 = Housing Choice Vouchers, 2016	0.168
	Percent of population living in poverty, 2016	0.167
	Average household income as a percent of local area median family income as defined by HUD, and adjusted for household size for Program 3 = Housing Choice Vouchers, 2016	0.166
	% of households with income below 50% of local area median family income,as defined by HUD, and adjusted for household size for Program 1 = Summary of All HUD Programs, 2015	-0.166
	% of households with income below 30% of local area median family income,as defined by HUD, and adjusted for household size for Program 3 = Housing Choice Vouchers, 2016	-0.164
	Average number of mentally unhealthy days reported in past 30 days (age-adjusted)	0.164
	Unemployment Rate for 2015	0.164
	Average household income as a percent of local area median family income as defined by HUD, and adjusted for household size for Program 1 = Summary of All HUD Programs, 2016	0.163
	Number of extreme heat days: Heat Index, 105°F, 2015	0.163
	Percentage of adults who are current smokers	0.163
	Birth Rates among Black women aged 15–19, 2014	0.163
	Percentile Percentage of persons below poverty estimate, 2018	0.163
	Average household income as a percent of local area median family income as defined by HUD, and adjusted for household size for Program 2 = Public Housing, 2015	0.162
	% of households who were in the program less than a year from the date of Picture snapshot for Program 3 = Housing Choice Vouchers, 2018	0.161
	% of households who were in the program less than a year from the date of Picture snapshot for Program 1 = Summary of All HUD Programs, 2017	0.160
	% of households with income below 50% of local area median family income,as defined by HUD, and adjusted for household size for Program 2 = Public Housing, 2017	-0.16
	Percentage of households that have income in the bracket of $20,000 or more yearly for Program 3 = Housing Choice Vouchers, 2018	-0.159
	Percentage of households that have income in the bracket of $5000-$9999 yearly for Program 1 = Summary of All HUD Programs, 2015	0.158
	Flag—the percentage of persons in institutionalized group quarters is in the 90th percentile(1 = yes, 0 = no), 2018	0.157
	% of households with 2 bedroom units for Program 5 = Project Based Sect. 8 = 202/PRAC, 2017	0.156
	Percentage of population with adequate access to locations for physical activity	-0.156
	Number of extreme heat events: Maximum Temperature, 90°F, 3 days minimum, 2014	0.156
	Birth Rates, Black women aged 18–19, 2014	0.155
	Average household income as a percent of local area median family income as defined by HUD, and adjusted for household size for Program 3 = Housing Choice Vouchers, 2015	0.155
	Average household income as a percent of local area median family income as defined by HUD, and adjusted for household size for Program 2 = Public Housing, 2016	0.154
	Number of extreme heat days: Maximum Temperature, 95°F, 2014	0.154
	% of households with 2 bedroom units for Program 5 = Project Based Sect. 8 = 202/PRAC, 2018	0.154
	% of households with income below 30% of local area median family income,as defined by HUD, and adjusted for household size for Program 2 = Public Housing, 2014	-0.153
	Average number of years a person can expect to live	-0.152
	Average household income as a percent of local area median family income as defined by HUD, and adjusted for household size for Program 3 = Housing Choice Vouchers, 2014	0.152
	% of households with income below 30% of local area median family income,as defined by HUD, and adjusted for household size for Program 3 = Housing Choice Vouchers, 2015	-0.150
Hispanic		
	Percentage of households than have income in the bracket of $10,000-$14,999 yearly for Program 2 = Public Housing, 2015	-0.167
	Percentage of households than have income in the bracket of $10,000-$14,999 yearly for Program 2 = Public Housing, 2016	-0.165
	Birth Rates – aged 15–17, 2014	0.160
	Percent High School Education, 2014	-0.157
	Percentage of households that have income in the bracket of $5000-$9999 yearly for Program 2 = Public Housing, 2017	0.152
	Percentage of households in which the older of the household head or spouse is 51 to 61 years of age for Program 3 = Housing Choice Vouchers, 2018	-0.152
Non-Hispanic White		
	Average number of years a person can expect to live	-0.188
	Years of potential life lost before age 75 per 100,000 population (age-adjusted)	0.183
	Sum of series for Socioeconomic theme, 2014^a^	0.181
	Percentage of adults aged 20 and over reporting no leisure-time physical activity	0.180
	Percent of population without health insurance, 2014	0.176
	Percentile Percentage of persons with no high school diploma(age 25 +) estimate, 2018	0.174
	Median Household Income, 2016	-0.171
	Percentage of adults who are current smokers	0.170
	Per Capita Personal Income, 2016	-0.169
	Percentage of households that have income in the bracket of $20,000 or more yearly for Program 5 = Project Based Sect. 8 = 202/PRAC, 2017	-0.167
	Percent of population living in poverty, 2016	0.167
	Percentage of adults aged 20 and above with diagnosed diabetes	0.166
	Percentage of adults ages 25–44 with some post-secondary education	-0.165
	Percentage of households that have income in the bracket of $20,000 or more yearly for Program 5 = Project Based Sect. 8 = 202/PRAC	-0.163
	Percentile percentage of civilian noninstitutionalized population with a disability	0.160
	Birth Rates, white, aged 40–54, 2014	-0.158
	Birth Rates, aged 15–19, 2014	0.158
	Median Household Income dollar, 2015	-0.157
	Percentage of households that have income in the bracket of $20,000 or more yearly for Program 3 = Housing Choice Vouchers	-0.157
	Percentage of adults reporting 14 or more days of poor physical health per month	0.156
	Percent Persons in Poverty, 2015	0.155
	Percentage of households that have income in the bracket of $20,000 or more yearly for Program 3 = Housing Choice Vouchers	-0.155
	Percentage of children enrolled in public schools that are eligible for free or reduced price lunch	0.154
	Average number of mentally unhealthy days reported in past 30 days (age-adjusted)	0.154
	Percentage of households that have income in the bracket of $20,000 or more yearly for Program 5 = Project Based Sect. 8 = 202/PRAC	-0.154
	Average total household income per year for Program 3 = Housing Choice Vouchers	-0.152
	Percentage of households that have income in the bracket of $20,000 or more yearly for Program 1 = Summary of All HUD Programs	-0.152
	Percentage of households that have income in the bracket of $20,000 or more yearly for Program 5 = Project Based Sect. 8 = 202/PRAC	-0.151
	Percentage of households that have income in the bracket of $20,000 or more yearly for Program 5 = Project Based Sect. 8 = 202/PRAC	-0.151
	Percent High School Education, 2014	-0.151
	Birth Rates, aged 18–19 years	0.151
	Average gross household contribution towards rent per month (includes payment toward rent and utilities) (Excludes zero values and missing values) for Program 3 = Housing Choice Vouchers	-0.150

*HUD* Housing and Urban Development, *PRAC* Project Rental Assistance Contract

^a^socioeconomic theme: below poverty, unemployed, income, no high school diploma

In domain-specific analyses of total population PRM (Table 4), an array of variables was found to be consistently correlated with overall PRM. For the social domain, various indicators of poverty, lack of access to food, and low educational level were among the strongest correlates. In the health domain, overall life expectancy, smoking (*r* = 0.246), lack of activity, and diabetes (*r* = 0.184) were associated with total population PRM. There was also a positive correlation with obesity (*r* = 0.179), firearm deaths (*r* = 0.174), rate of AIDS among men who have sex with men (*r* = 0.168), and an inverse correlation with heavy drinking (*r* = -0.168). For the built environment, healthy food environment (*r* = -0.228), several indicators of income related to housing programs, and percentage of mobile-homes (*r* = 0.155) were correlated with PRM. For the natural and policy environment, most correlations were below our cut-offs. The strongest correlations for the natural environment were all with extreme heat (*r* = 0.13–0.19). In the policy environment, the only correlations greater than *r* = 0.1 were with programs to address child poverty (free school lunch eligibility, *r* = 0.184). Domain-specific analyses of correlations with maternal racial/ethnic specific PRM are presented in supplemental tables S2-S5.

**Table 4:** Domain-specific factors most strongly associated with national 4-year total population pregnancy-related mortality ratio per 100,000 live births (2015–2018). Correlations are with respect to the relevant PRM ratio variable

**Social environment**	
Percent of population living in poverty, 2016	0.241
Percentage of population who lack adequate access to food	0.232
Percent Persons in Poverty, 2015	0.231
Sum of series for Socioeconomic theme^a^	0.229
Median Household Income, 2016	-0.219
Percentile Percentage of persons with no high school diploma(age 25 +) estimate, 2018	0.200
Sum of series for Household Composition theme, 2014	0.196
Percentage of children that live in a household headed by single parent	0.193
Percentage of population that is non-Hispanic Black or African American	0.189
Percentile percentage of civilian noninstitutionalized population with a disability estimate, 2018	0.187
Percentage of adults ages 25–44 with some post-secondary education	-0.184
Per Capita Personal Income, 2016	-0.182
Median Household Income in dollars, 2015	-0.180
Percent High School Education percent, 2014	-0.163
Ratio of household income at the 80th percentile to income at the 20th percentile	0.159
Percentile percentage of single parent households with children under 18 estimate, 2018	0.153
Flag—the percentage of single parent households is in the 90th percentile (1 = yes,0 = no), 2018	0.153
**Health and health care environment**	
Years of potential life lost before age 75 per 100,000 population (age-adjusted)	0.254
Average number of years a person can expect to live	-0.249
Percentage of adults who are current smokers	0.246
Percentage of adults aged 20 and over reporting no leisure-time physical activity	0.237
Births unmarried women, non-Hispanic, 2014	0.220
Percentage of adults reporting 14 or more days of poor physical health per month	0.211
Birth Rates, aged 15–19, 2014	0.201
Percent Living in Poverty percent, 2015	0.192
Percentage of adults who report fewer than 7 h of sleep on average	0.192
Birth Rates, aged 18–19, 2014	0.190
Average number of mentally unhealthy days reported in past 30 days (age-adjusted)	0.185
Percentage of adults aged 20 and above with diagnosed diabetes	0.184
Median Household Income in dollars, 2015	-0.180
Percentage of the adult population (age 20 and older) that reports a body mass index (BMI) greater than or equal to 30 kg/m^2^	0.179
Percent of population without health insurance	0.178
Number of deaths due to firearms per 100,000 population	0.174
Percentage of adults reporting binge or heavy drinking	-0.168
Rate of HIV among MSM, 2018	0.163
Age-adjusted rate of death from Heart Attack among persons 35 and older per 100,000 population, 2015	0.160
Age-adjusted rate of death from Heart Attack among persons 35 and older per 100,000 population, 2014	0.158
Birth Rates, aged 40–54, white, 2014	-0.157
Standardized Per Capita Medicare Cost, 2014	0.155
Number of newly diagnosed chlamydia cases per 100,000 population	0.154
Preventable Hospital Stays Rate, 2015	0.151
Rate of hospital stays for ambulatory-care sensitive conditions per 100,000 Medicare enrollees	0.150
**Built environment**	
Index of factors that contribute to a healthy food environment, from 0 (worst) to 10 (best)	-0.228
Average gross household contribution towards rent per month (includes payment toward rent and utilities) (Excludes zero values and missing values) for Program 3 = Housing Choice Vouchers, 2017	-0.186
Percentage of households that have income in the bracket of $20,000 or more yearly for Program 5 = Project Based Sect. 8 = 202/PRAC, 2018	-0.185
Average gross household contribution towards rent per month (includes payment toward rent and utilities) (Excludes zero values and missing values) for Program 5 = Project Based Sect. 8 = 202/PRAC, 2017	-0.181
Percentage of households that have income in the bracket of $5000-$9999 yearly for Program 2 = Public Housing, 2017	0.180
Percentage of households that have income in the bracket of $20,000 or more yearly for Program 5 = Project Based Sect. 8 = 202/PRAC, 2018	-0.179
Percentage of households that have income in the bracket of $5000-$9999 yearly for Program 2 = Public Housing, 2017	0.179
Percentage of households that have income in the bracket of $5000-$9999 yearly for Program 1 = Summary of All HUD Programs, 2018	0.177
% of the population below poverty level in the census tract where HUD assisted families reside (Census 2010 designation) for Program 3 = Housing Choice Vouchers, 2018	0.175
Percentage of households that have income in the bracket of $5000-$9999 yearly for Program 2 = Public Housing, 2015	0.175
Percentage of households that have income in the bracket of $20,000 or more yearly for Program 5 = Project Based Sect. 8 = 202/PRAC, 2016	-0.170
Percentage of households that have income in the bracket of $20,000 or more yearly for Program 5 = Project Based Sect. 8 = 202/PRAC, 2014	-0.168
Percentage of households that have income in the bracket of $5000-$9999 yearly for Program 1 = Summary of All HUD Programs, 2015	0.167
Percentage of households that have income in the bracket of $5000-$9999 yearly for Program 2 = Public Housing, 2016	0.166
Percentage of households in which the race of the head of household is Black and the ethnicity is not Hispanic for Program 1 = Summary of All HUD Programs, 2018	0.165
Average gross household contribution towards rent per month (includes payment toward rent and utilities) (Excludes zero values and missing values) for Program 2 = Public Housing, 2014	-0.164
Percentages of households headed by female for Program 3 = Housing Choice Vouchers, 2015	0.161
Average gross household contribution towards rent per month (includes payment toward rent and utilities) (Excludes zero values and missing values) for Program 3 = Housing Choice Vouchers, 2015	-0.156
Average gross household contribution towards rent per month (includes payment toward rent and utilities) (Excludes zero values and missing values) for Program 2 = Public Housing, 2017	-0.155
Percent of mobile-home housing units, 2016	0.155
Percentage of households that have income which comes in the bracket of $1-$4999 yearly for Program 5 = Project Based Sect. 8 = 202/PRAC, 2018	0.153
Average gross household contribution towards rent per month (includes payment toward rent and utilities) (Excludes zero values and missing values) for Program 2 = Public Housing, 2016	-0.152
Percentage of households that have income in the bracket of $20,000 or more yearly for Program 3 = Housing Choice Vouchers, 2018	-0.152
Percentage of population with adequate access to locations for physical activity	-0.151
Average total household income per year for Program 1 = Summary of All HUD Programs, 2017	-0.151
Average gross household contribution towards rent per month (includes payment toward rent and utilities) (Excludes zero values and missing values) for Program 2 = Public Housing, 2015	-0.150
Percentages of households headed by female for Program 1 = Summary of All HUD Programs, 2016	0.150
**Natural environment**	
Number of extreme heat days: Heat Index, 105°F, 2015	0.192
Number of extreme heat days: Heat Index, 95°F, 2014	0.169
Number of extreme heat events: Heat Index, 95°F, 3 days minimum, 2014	0.164
Number of extreme heat events: Heat Index, 100°F, 3 days minimum, 2016	0.156
**Policy environment**^**a**^	
Free school lunch eligibility (percent), 2014	0.184
National School Lunch Program participants (% pop), 2014	0.178
Percentage of children enrolled in public schools that are eligible for free or reduced price lunch	0.176
School Breakfast Program participants (% pop), 2014	0.166

^a^After removal of the autocorrelates and margin of error variables, only 8 variables remained in this domain

In the unsupervised analysis, two paracliques (threshold 15, glom 3, *n* = 77 and *n* = 12 variables, Table S5) contained the overall and race/ethnic-specific PRM variables. They also contained several indicators of health care availability, crime arrests (mostly for minor crimes such as bookmaking, curfew, and numbers & lottery, but also embezzlement and offenses against children and adults), population indicators such as birth rates among adolescents, some factors related to water use, and poverty and income inequality. The paraclique containing Hispanic PRM contained variables related to poverty, public housing, and Medicare quality.

In the Bayesian analysis (Table 5), within the factors most correlated with PRM (*r* > 0.15), the upstream indicators of overall PRM was access to healthy food. Within the built environment, two factors were indicators of overall PRM: access to healthy food; in the health domain, years of potential life lost, proportion of adults reporting no leisure-time physical activity, heart attacks, and chlamydia were most highly correlated; in the natural environment, extreme heat days; and in the social environment, poverty and summary low SES had the highest correlations with overall PRM.

**Table 5:** Summary of direct predictors based on Bayesian network analysis, pregnancy-related mortality, by domain and race/ethnicity

domain	race/ethnicity	PHE factor
overall	all	Index of factors that contribute to a healthy food environment, from 0 (worst) to 10 (best)
built	all	Indicator of access to Healthy Foods—0 is worst, 10 is best
health	all	Years of potential life lost before age 75 per 100,000 population (age-adjusted)
		Percentage of adults aged 20 and over reporting no leisure-time physical activity.
		Age-adjusted rate of death from Heart Attack among persons 35 and older per 100,000 population, 2015
		Number of newly diagnosed chlamydia cases per 100,000 population
natural	all	Number of Extreme Heat days: Heat Index, 105°F, 2015
policy	all	National School Lunch Program participants (% pop), 2014
		Free school lunch eligibility (percent), 2014
social	all	Percent of population living in poverty, 2016
		Sum of series for Socioeconomic theme, 2014*
overall	Black	Percentage of households in which the older of the household head or spouse is 24 or less years of age for Program 3 = Housing Choice Vouchers, 2014
		Daily county-level population weighted spectral irradiance at local solar noon time at 305 nm. (Jul), 2015
		Flag—the percentage of persons in institutionalized group quarters is in the 90th percentile(1 = yes, 0 = no), 2018
		% of households with 2 bedroom units for Program 5 = Project Based Sect. 8 = 202/PRAC, 2018
built	Black	Average Household Income as a % of Local Area Median Family Income as defined by HUD, and Adjusted for Household Size for Program 2 = Public Housing, 2014
		% of Households in which the Older of the Household Head or Spouse is 24 or less Years of Age for Program 3 = Housing Choice Vouchers, 2014
		% of households with 2 bedroom units for Program 5 = Project Based Sect. 8 = 202/PRAC, 2018
health	Black	Births to unmarried women, white, non-Hispanic, 2014
		Percentage of adults who report fewer than 7 h of sleep on average
natural	Black	Domestic, publicly supplied per capita use, in gallons/day, 2015
policy	Black	None
social	Black	Percent of population living in poverty, 2016
overall	Hispanic	Percentage of households that have income in the bracket of $5000-$9999 yearly for Program 2 = Public Housing, 2016
		Percent High School Education percent, 2014
built	Hispanic	Percentage of households that have income in the bracket of $5000-$9999 yearly for Program 2 = Public Housing, 2017
health	Hispanic	Birth Rates, ages 15-17, 2014
natural	Hispanic	None
policy	Hispanic	None
social	Hispanic	Percent High School Education, 2014
overall	White	Percentage of adults age 20 and over reporting no leisure-time physical activity
		Average annual percent of diabetic Medicare enrollees age 65-75 having eye examination: White: Rate, 2015
		Percentage of households that have income in the bracket of $20,000 or more yearly for Program 3=Housing Choice Vouchers, 2018
built	White	Percentage of households that have income in the bracket of $20,000 or more yearly for Program 5 = Project Based Sect. 8 = 202/PRAC, 2017
health	White	Birth Rates, aged 40-54, 2014
		Average annual percent of diabetic Medicare enrollees aged 65-75 having eye examination: White: Rate, 2015
natural	White	Daily County-Level Population Weighted Spectral Irradiance at Local Solar Noon Time at 310 nm. (Mar)
policy	White	Percentage of children enrolled in public schools that are eligible for free or reduced price lunch
social	White	Sum of series for Socioeconomic theme, 2014*
		Per Capita Personal Income, 2016

*socioeconomic theme: below poverty, unemployed, income, no high school diploma

### Study limitations

A limitation of the data used in this study is the relatively small number of PRM cases. Only county equivalents with at least 1000 live births were considered in order to increase rate stability. However, this limited the number of counties that could be examined, particularly for race/ethnicity-specific analyses, and results were still vulnerable to outliers. The outcome could not be analyzed at a lower level, such as neighborhood, due to the rarity of the event and the need to harmonize across data sources. This analysis relied on death records alone to identify cases of PRM. In addition, the method used to identify cases of PRM was limited to information drawn from death records along (ICD-10 code for underlying cause of death). This differs from the CDC’s identification of PRM, which utilizes multiple sources of data and expert reviews in order to determine pregnancy-relatedness in each case. As such, comparisons between these measures cannot be made. Temporal or lagged effects were not considered, because of the approach used to determine PRM (combined 4-year ratio). Finally, while the PHE dataset includes over 62,000 variables to capture area-level features of built, policy, social, and natural environments, the PHE may still fail to capture the totality of potential forces driving PRM in this country. For instance, while a few measures of residential segregation and income inequality are among the PHE variables (but did not turn out to be strong predictors relative to other factors), other measures of structural racism such as racial inequities in education, income, homeownership, or criminal justice [37, 38]; or historical policies like redlining [39] are not included. The policies included in the exposome are relatively limited, and do not, for instance, include those around drugs, sentencing, abortion, or Medicaid eligibility. Causality cannot be explored within an ecological analysis, and inferences cannot be drawn about individual-level associations (it is not known, for example, whether women who died were in poverty or lacked health insurance).

Relying solely on correlation to determine autocorrelates may eliminate potentially highly correlated independent variables from the study. The reduction in autocorrelates, however, produced clearer results. When selecting top correlates to PRM, choosing the 20 most correlated variables may leave out potentially revealing associations. Twenty variables struck a reasonable balance between too many to visualize and too few for exploratory analysis. While dense subgraphs containing PRM via paraclique were found, there is no guarantee that all latent networks that include PRM were detected. This be due to the low overall correlation between PRM and the PHE, requiring a threshold of 0.15. The low cluster thresholds indicate highly subtle variable associations. Finally, all results in this study rely on the details of the methods used and different choices of parameters might affect the results (not unlike alpha = 0.05 or 0.10 for choosing statistical predictors). For this reason, when available, other than for paracliques, we used default software parameter settings for all methods used to aid reproducibility.

## Discussion

In this high-dimensional analysis of over 3900 potential predictors, years of potential life lost was found to be the single, most consistent predictor of total population PRM. The number of maternal deaths is very small relative to other contributors to total years of potential life lost [40], so reverse causality is an unlikely cause of this finding. Since maternal deaths are unusual in occurring in young, often otherwise healthy women, it is not necessarily obvious that they should correlate with overall indicators of mortality, which are driven primarily by older sick people. Reproductive and pregnancy health is often treated as separate, or secondary, to other aspects of health, but this analysis suggests that the major risk factors driving maternal mortality are the same as those driving health more generally. Other related factors by race/ethnicity included overall life expectancy, poverty, smoking, lack of physical activity, and lack of access to healthy food. This paints a clear picture of maternal death as part and parcel of an adverse, social environmental context, rather than caused by a limited, proximal set of risk factors. Maternal deaths are highest in areas where life expectancy is already low. While poverty is widely associated with health [41], it is still striking that the cluster of poverty-related factors rather than potentially more proximal factors such as obstetric services or hypertension rates remain the strongest predictors even after examining thousands of other exposures. The complete list of variables for the built environment domain, for instance, started with about 8700 variables and includes factors like land use; commute time; crowding and density, and rurality; housing age and quality; heating type; amount of housing units, size, and availability; and number of private nonfarm and farm establishments and their characteristics. However, the strongest correlations with PRM within that domain described the social characteristics of the people living in that built environment. Many of the variables in the PHE are related, interconnected, and may be linked to mortality via multiple, multilevel, and complex pathways. In such a case identifying individual risk factors may be less useful than capturing the broader context, represented by multiple indicators, which was accomplished in this analysis.

Race/ethnicity-specific analyses confirmed this overall pattern of interconnected social factors and general health indicators being the strongest predictors of PRM, with identified factors indicating socioeconomic status (education, unemployment) or general population health (adults in fair/poor health, overall life expectancy) as the most important correlates with PRM. While individual variables differed by race/ethnicity, overall, they were part of a larger constellation representing social deprivation (low education, low income, unemployment, lack of health insurance). This finding supports the notion that racism, not race, underlies racial inequities in maternal mortality [11] as with other population health inequities [42, 43]. Historical and contemporary racism restricts access to health-promoting resources such as income, wealth, and education, which results in disproportionate morbidity and mortality [44, 45]. Finally, a few specific health conditions of the population also were identified (diabetes, obesity, heart attacks), which is consistent with the high proportion of pregnancy-related deaths from underlying cardiovascular disease conditions [46].

The graph theoretical methods used in this analysis have several advantages, and we examine our results to determine whether such advantages were realized. First, these methods scale to extreme numbers of variables without issues of multiple comparisons. In this case, several thousand variables and their associations were studied simultaneously. Second, the methods used to elucidate intercorrelated subgroups (paracliques) can provide new insights when compared to traditional methods that can only study associations between variable pairs. In this analysis, various latent associations were thereby identified. Third, these methods allow for the discovery of novel associations or entire groups of factors that are highly intercorrelated. While it is not a surprise to see associations of PRM with lack of physical activity, unhealthy food environment, or low mental health, our first hypotheses might have concerned access to obstetric care or underlying medical causes such as hypertension instead. Thus, the methods employed in the current study fulfilled the goal of identifying novel associations. Finally, use of these methods in the PHE specifically allowed for finding joint effects of the physical and social environments. While most associations identified were from the social and health care domains, some physical environment factors, particularly heat, were also identified as important.

By comparison, our previous work used hypothesis-driven modeling, and found associations between maternal mortality and income inequality [47], levels of violent crime [48], and access to maternity care [49]. These more standard analyses concentrated on specific factors of interest with control for covariates, a model that better supports causal inference. That research, however, was limited to specific social factors, analyzed one at a time. Attempting this for thousands of predictors would be plagued by multiple comparisons, while examining all possible variable combinations would quickly become computationally intractable. The graph theoretical methods we have employed are often best viewed as hypothesis generators. With ecological data especially, they can provide a solid basis for more detailed study incorporating multi-level data, confounders, and mechanistic analysis.

This is an exploratory, ecological study of aggregate-level population data. The results are broadly consistent with a recent hypothesis-based investigation of county-level predictors of PRM in the U.S. which found that county-level unemployment and food insecurity were risk factors and higher income, education, and owner-occupied household were protective factors [9]. Many public health measures are administered at the county level, and for many of the variables included in the Public Health Exposome, existing literature identifies the county level as a relevant geographic resolution for their association with health outcomes [50, 51]. The processes underlying PRM are multi-level, and our focus on the county level does not negate the importance of considering state or more localized levels as well. Indeed, existing evidence identifies state-level contexts, structures, and policies that contribute to the vastly different PRM rates that occur across states [47], but these analyses mask the variation occurring within states. The analysis also pulled out the strongest correlations, not necessarily those that are causal. For instance, food environment could be causally related to maternal death, or it could simply be strongly correlated with income and other health-promoting resources. Other factors, such as rate of HIV among men who have sex with men, are likely a combination of social indicator and indicator of related health factors, such as access to contraception and sexual health services. Most of the data used are national, so should be measured consistently across counties. Ecological studies can be particularly vulnerable to cross-level confounding and interactions [52, 53]; the results of our study are consistent with what is known about individual-level associations, so we think the more important concern is to address multi-level influences and the interactions among effects occurring at different levels.

Strengths of this study include the use of a nationwide, 2015–2018, maternal mortality data set and the wide-ranging environmental and health risk factors represented by the PHE that were considered in the analysis. As can be seen in the tables, the most predictive factors were drawn from a range of databases, and did not necessarily center on a single one, highlighting the value of the full exposome database. If all the predictive results came from a single data source, we should more effectively and efficiently focus our efforts on that source. As the factors we found to be most important are drawn from several of the dozens of databases included in the PHE, it is serving its purpose of describing the range of exposures that may be influencing public health.

## Conclusions

Recent years have seen a heightened concern about pregnancy-related mortality in general and disparities in PRM in particular. The examination of 1591 potential associated variables in our analyses did not find unusual or novel risk factors; rather, it confirms that this issue is not separate from the United States’ socioeconomic inequalities and general health problems, but is embedded in them. Study findings contribute to the growing body of evidence documenting the impact of adverse social and environmental factors on maternal death [47, 54, 55]. From a methodological standpoint, we continue to develop “big data” methods that can deal with social and environmental factors as rigorously and completely as the methods for addressing genomics or transcriptomics data. Scalable combinatorial methods have achieved impressive successes in the analysis of high throughput, high fidelity -omics data. In domains such as social science and public health, however, their reach is sometimes limited by data quality and type. Prohibitive levels of noise can occur in many forms, including data inaccuracies, collinearities, conflicts, and uneven sampling. This motivates the sustained development of robust, noise-resilient methods such as the paraclique algorithm and effective feature selection and preprocessing techniques such as MDS-90. From a content standpoint, efforts to reduce maternal mortality must expand beyond individual and clinical risk factors to include the effect of harmful social and environmental contexts, with a particular focus on the experience of women from different racial and ethnic groups, and those who are socio-economically disadvantaged. Further investigation of the intricate links between environmental exposures and maternal death is crucial to elucidate the mechanisms and pathways through which contextual factors contribute to the detrimental health effects of exposed individuals.

## Supplementary Information

**Additional file 1.**

## Data Availability

Maternal mortality data were acquired from NCHS (https://www.cdc.gov/nchs/data/data_access_and_resources_booklet_web.pdf). The public health exposome is compiled from publicly-available data sources and maintained by Meharry Medical College Health Disparities Research Center of Excellence. Paul Juarez can be contacted for more information (pjuarez@mmc.edu).

## Abbreviations

MM: Maternal mortality
MMR: Maternal mortality ratio
NCHS: National Center for Health Statistics
NH: Non-Hispanic
PHE: Public health exposome
PRM: Pregnancy-related mortality
YPLL: Years of potential life lost
