# Supplementary material for: The public health exposome and pregnancy-related mortality in the United States: a high-dimensional computational analysis
Source: BMC Public Health. 2022 Nov 17;22:2097. doi: 10.1186/s12889-022-14397-x (PMC9670647; doi:10.1186/s12889-022-14397-x)

Table S1a. Summary statistics for the listed variables over all 1709 counties included in the present study.

| **Variable** | **Mean** | **std** | **Min** | **25^th^ Percentile** | **50^th^ Percentile** | **75^th^ Percentile** | **Max** |
| --- | --- | --- | --- | --- | --- | --- | --- |
| Total Population Maternal Mortality Rate | 35.6 | 41.7 | 0 | 0 | 24 | 56.3 | 293.3 |
| Median Income (USD) | 51258.6 | 13283.3 | 23968 | 42301 | 48864 | 56975 | 125900 |
| Percent Below Poverty Line | 16.1% | 6.0% | 3.7% | 12.0% | 15.7% | 19.4% | 47.1% |
| Percent NHAA | 10.0% | 13.4% | 0.1% | 1.3% | 4.2% | 12.8% | 76.6% |
| Percent Hispanic | 10.5% | 13.7% | 0.7% | 2.8% | 5.5% | 11.6% | 96.4% |
| Percent with High School Diploma | 85.8% | 6.3% | 51.3% | 82.3% | 87.1% | 90.3% | 97.7% |
| Unemployment Rate | 5.6 | 1.7 | 2.2 | 4.4 | 5.3 | 6.4 | 24 |
| Population | 177488 | 428176.9 | 10065 | 35556 | 60853 | 152538 | 10163507 |
| Percent Under 18 | 24% | 3.1% | 9.1% | 22.0% | 23.9% | 25.6% | 40.1% |
| Percent Over 65 | 14.2% | 3.4% | 5.6% | 12.1% | 14.2% | 16.1% | 43.4% |
| Birth Rates | 1178.8 | 226.6 | 379.3 | 1036.2 | 1158.7 | 1288.2 | 2888.8 |
| Percent Uninsured Under 65 | 10.6% | 4.8% | 2.3% | 6.8% | 9.7% | 13.5% | 33.7% |
| Percent Smokers | 17.4% | 3.4% | 5.9% | 15.0% | 17.3% | 19.5% | 41.5% |

Table S1b. Summary statistics for the listed variables over all 1581 counties excluded from the present study.

| **Variable** | **Mean** | **std** | **Min** | **25^th^ Percentile** | **50^th^ Percentile** | **75^th^ Percentile** | **Max** |
| --- | --- | --- | --- | --- | --- | --- | --- |
| Total Population Maternal Mortality Rate | 46.1 | 149.6 | 0 | 0 | 0 | 0 | 3030.3 |
| Median Income (USD) | 45396.8 | 10354.7 | 0 | 38105 | 44119 | 51219 | 122092 |
| Percent Below Poverty Line | 18.4% | 10.2% | 1.8% | 11.7% | 16.1% | 21.7% | 63.8% |
| Percent NHAA | 7.8% | 15.2% | 0.0% | 0.5% | 0.9% | 4.8% | 85.4% |
| Percent Hispanic | 8.7% | 14.0% | 0.6% | 2.1% | 3.4% | 7.1% | 94.6% |
| Percent with High School Diploma | 85.0% | 7.0% | 46.3% | 80.0% | 86.6% | 90.6% | 98.4% |
| Unemployment Rate | 6 | 3.1 | 1.8 | 3.8 | 5.35 | 7.2 | 24.3 |
| Population | 17127.5 | 99943.6 | 88 | 5841.8 | 10541 | 16429.5 | 2648771 |
| Percent Under 18 | 22.8% | 3.5% | 0.0% | 20.8% | 22.7% | 24.4% | 41.6% |
| Percent Over 65 | 17.9% | 4.2% | 3.5% | 15.1% | 17.7% | 20.6% | 34% |
| Birth Rates | 1095.6 | 251.2 | 90.4 | 943.7 | 1084.4 | 1213.8 | 2592.1 |
| Percent Uninsured Under 65 | 12.5% | 5.3% | 3.0% | 8.3% | 11.6% | 15.8% | 32.7% |
| Percent Smokers | 17.6% | 3.9% | 7.4% | 14.8% | 16.6% | 19.9% | 41.0% |

Table S2. Built environment factors most strongly associated with national 4-year total population pregnancy-related mortality ratio per 100,000 live births (2015-2018), stratified by race/ethnicity

| **Maternal race/ethnicity** | **PHE Built Environnent Domain variable description** | **Correlation** |
| --- | --- | --- |
| Non-Hispanic Black pregnancy-related mortality | |  |
|  | Percentage of households in which the older of the household head or spouse is 24 or less years of age for Program 3=Housing Choice Vouchers | 0.194 |
|  | Average household income as a percent of local area median family income as defined by HUD, and adjusted for household size for Program 2=Public Housing | 0.184 |
|  | Percentage of households in which the older of the household head or spouse is 24 or less years of age for Program 3=Housing Choice Vouchers | 0.180 |
|  | % of households who were in the program less than a year from the date of Picture snapshot for Program 3=Housing Choice Vouchers | 0.168 |
|  | Average household income as a percent of local area median family income as defined by HUD, and adjusted for household size for Program 3=Housing Choice Vouchers | 0.166 |
|  | % of households with income below 50% of local area median family income, as defined by HUD, and adjusted for household size for Program 1=Summary of All HUD Programs | -0.166 |
|  | % of households with income below 30% of local area median family income,as defined by HUD, and adjusted for household size for Program 3=Housing Choice Vouchers | -0.164 |
|  | Average household income as a percent of local area median family income as defined by HUD, and adjusted for household size for Program 1=Summary of All HUD Programs | 0.163 |
|  | Average household income as a percent of local area median family income as defined by HUD, and adjusted for household size for Program 2=Public Housing | 0.162 |
|  | % of households who were in the program less than a year from the date of Picture snapshot for Program 3=Housing Choice Vouchers | 0.161 |
|  | % of households with income below 50% of local area median family income,as defined by HUD, and adjusted for household size for Program 2=Public Housing | -0.160 |
|  | % of households who were in the program less than a year from the date of Picture snapshot for Program 1=Summary of All HUD Programs | 0.160 |
|  | Percentage of households that have income in the bracket of $20000 or more yearly for Program 3=Housing Choice Vouchers | -0.159 |
|  | Percentage of households that have income in the bracket of $5000-$9999 yearly for Program 1=Summary of All HUD Programs | 0.158 |
|  | % of households with 2 bedroom units for Program 5=Project Based Section 8=202/PRAC | 0.156 |
|  | Percentage of population with adequate access to locations for physical activity. | -0.156 |
|  | Average household income as a percent of local area median family income as defined by HUD, and adjusted for household size for Program 3=Housing Choice Vouchers | 0.155 |
|  | Average household income as a percent of local area median family income as defined by HUD, and adjusted for household size for Program 2=Public Housing | 0.154 |
|  | % of households with 2 bedroom units for Program 5=Project Based Section 8=202/PRAC | 0.154 |
|  | % of households with income below 30% of local area median family income,as defined by HUD, and adjusted for household size for Program 2=Public Housing | -0.153 |
|  | Average household income as a percent of local area median family income as defined by HUD, and adjusted for household size for Program 3=Housing Choice Vouchers | 0.152 |
|  | % of households with income below 30% of local area median family income,as defined by HUD, and adjusted for household size for Program 3=Housing Choice Vouchers | -0.150 |
| Hispanic pregnancy-related mortality | |  |
|  | Percentage of households than have income in the bracket of $10000-$14999 yearly for Program 2=Public Housing | -0.167 |
|  | Percentage of households than have income in the bracket of $10000-$14999 yearly for Program 2=Public Housing | -0.165 |
|  | Percentage of households that have income in the bracket of $5000-$9999 yearly for Program 2=Public Housing | 0.152 |
|  | Percentage of households in which the older of the household head or spouse is 51 to 61 years of age for Program 3=Housing Choice Vouchers | -0.152 |
| Non-Hispanic White pregnancy-related mortality | |  |
|  | Percentage of households that have income in the bracket of $20000 or more yearly for Program 5=Project Based Section 8=202/PRAC | -0.167 |
|  | Percentage of households that have income in the bracket of $20000 or more yearly for Program 5=Project Based Section 8=202/PRAC | -0.163 |
|  | Percentage of households that have income in the bracket of $20000 or more yearly for Program 3=Housing Choice Vouchers | -0.157 |
|  | Percentage of households that have income in the bracket of $20000 or more yearly for Program 3=Housing Choice Vouchers | -0.155 |
|  | Percentage of households that have income in the bracket of $20000 or more yearly for Program 5=Project Based Section 8=202/PRAC | -0.154 |
|  | Average total household income per year for Program 3=Housing Choice Vouchers | -0.152 |
|  | Percentage of households that have income in the bracket of $20000 or more yearly for Program 1=Summary of All HUD Programs | -0.152 |
|  | Percentage of households that have income in the bracket of $20000 or more yearly for Program 5=Project Based Section 8=202/PRAC | -0.151 |
|  | Average gross household contribution towards rent per month (includes payment toward rent and utilities) (Excludes zero values and missing values) for Program 3=Housing Choice Vouchers | -0.150 |

Table S3. Health environment factors most strongly associated with national 4-year total population pregnancy-related mortality ratio per 100,000 live births (2015-2018), stratified by race/ethnicity.

| **Maternal race/ethnicity** | **PHE Health Environnent Domain variable description** | **Correlation** |
| --- | --- | --- |
| Non-Hispanic Black pregnancy-related mortality | |  |
|  | Births unmarried women, aged 18-54, non-Hispanic, 2014 | -0.206 |
|  | Percentage of adults who report fewer than 7 hours of sleep on average. | 0.192 |
|  | Percentage of adults reporting 14 or more days of poor physical health per month. | 0.177 |
|  | Average number of mentally unhealthy days reported in past 30 days (age-adjusted). | 0.164 |
|  | Percentage of adults who are current smokers. | 0.163 |
|  | Birth Rates, aged 15-19, 2014 | 0.163 |
|  | Birth Rates, aged 18-19, 2014 | 0.155 |
|  | Average number of years a person can expect to live. | -0.152 |
| Hispanic pregnancy-related mortality | |  |
|  | Birth Rates, aged 15-17 | 0.160 |
| Non-Hispanic White pregnancy-related mortality | |  |
|  | Average number of years a person can expect to live. | -0.188 |
|  | Years of potential life lost before age 75 per 100,000 population (age-adjusted). | 0.183 |
|  | Percentage of adults age 20 and over reporting no leisure-time physical activity. | 0.180 |
|  | Percent of population without health insurance. | 0.176 |
|  | Percentage of adults who are current smokers. | 0.170 |
|  | Percentage of adults aged 20 and above with diagnosed diabetes. | 0.166 |
|  | Birth Rates, aged 40-54, white, 2014 | -0.158 |
|  | Birth Rates, aged 15-19, 2014 | 0.158 |
|  | Median Household Income dollar, 2015 | -0.157 |
|  | Percentage of adults reporting 14 or more days of poor physical health per month. | 0.156 |
|  | Average number of mentally unhealthy days reported in past 30 days (age-adjusted). | 0.154 |
|  | Percent High School Education percent, 2014 | -0.151 |
|  | Birth Rates, aged 18-19, 2014 | 0.151 |

Table S4. Social and policy environment factors most strongly associated with national 4-year total population pregnancy-related mortality ratio per 100,000 live births (2015-2018), stratified by race/ethnicity

| **Maternal race/ethnicity** | **PHE Health Environnent Domain variable description** | **Correlation** |
| --- | --- | --- |
|  | **Social** |  |
| Non-Hispanic Black pregnancy-related mortality | |  |
|  | Average grade level performance for 3rd graders on math standardized tests | -0.171 |
|  | Sum of series for Socioeconomic theme | 0.169 |
|  | Percent of population living in poverty. | 0.167 |
|  | Unemployment Rate for 2015 | 0.164 |
|  | Percentile Percentage of persons below poverty estimate | 0.163 |
|  | Flag - the percentage of persons in institutionalized group quarters is in the 90th percentile(1 = yes, 0 = no) | 0.157 |
| Hispanic pregnancy-related mortality | | |
|  | Percent High School Education percent, 2014 | -0.157 |
| Non-Hispanic White pregnancy-related mortality | |  |
|  | Sum of series for Socioeconomic theme | 0.181 |
|  | Percentile Percentage of persons with no high school diploma(age 25+) estimate | 0.174 |
|  | Median Household Income, 2016 | -0.171 |
|  | Per Capita Personal Income, 2016 | -0.169 |
|  | Percent of population living in poverty. | 0.167 |
|  | Percentage of adults ages 25-44 with some post-secondary education. | -0.165 |
|  | Percentile percentage of civilian noninstitutionalized population with adisability estimate | 0.160 |
|  | Median Household Income dollar, 2015 | -0.157 |
|  | Percent Persons in Poverty, 2015 | 0.155 |
|  | Percent High School Education percent, 2014 | -0.151 |
|  | **Policy** |  |
| Black | none r>.15 |  |
| Hispanic | none r>.15 |  |
| White | Percentage of children enrolled in public schools that are eligible for free or reduced price lunch | 0.154 |

*Themes: socioeconomic, household composition and disability, minority status and language, housing and transportation

Table S5. Natural environment factors most strongly associated with national 4-year total population pregnancy-related mortality ratio per 100,000 live births (2015-2018), stratified by race/ethnicity

| **Maternal race/ethnicity** | **PHE Built Environnent Domain variable description** | **Correlation** |
| --- | --- | --- |
| Non-Hispanic Black pregnancy-related mortality | |  |
|  | Domestic, publicly supplied per capita use, in gallons/day [DO-PSDel/PS-TOPop], 2015 | 0.206 |
|  | Daily county-level population weighted spectral irradiance at local solar noon time at 305 nm. (Jul) | 0.180 |
|  | Number of extreme heat days: Maximum Temperature, 90°F | 0.174 |
|  | Number of extreme heat days: Heat Index, 105°F | 0.170 |
|  | Number of extreme heat days: Heat Index, 105°F | 0.163 |
|  | Number of extreme heat events: Maximum Temperature, 90°F, 3 days minimum | 0.156 |
|  | Number of extreme heat days: Maximum Temperature, 95°F | 0.154 |
| Hispanic pregnancy-related mortality | | none r>0.15 |
| Non-Hispanic White pregnancy-related mortality | | none r>0.15 |

Table S6. Paracliques containing PRM variables (threshold 15, glom 3)

| # Hosp W/Rural Health Clinic, 2016 |
| --- |
| # Rural Health Clinics, 2015 |
| % Change WIC-authorized stores, 08-09 |
| % Medcre Advantage Penetration, 2015 |
| % of households with income below 50% of local area median family income, as defined by HUD, and adjusted for household size for Program 3=Housing Choice Vouchers, 2014 |
| % of households with income below 50% of local area median family income, as defined by HUD, and adjusted for household size for Program 3=Housing Choice Vouchers, 2017 |
| 4-year NH Black pregnancy-related mortality ratio per 100,000 live births where at least 10 deaths (2015-2018) |
| 4-year NH White pregnancy-related mortality ratio per 100,000 live births where at least 10 deaths (2015-2018) |
| 4-year total population pregnancy-related mortality ratio per 100,000 live births where at least 10 deaths (2015-2018) |
| Birth Rates, <18 years, white, 2014 |
| Birth Rates, 15-19, black, 2014 |
| Birth Rates, <18, non-white, 2014 |
| Birth Rates, < 18, American Indian, 2014 |
| Birth Rates, 40-54, American Indian, 2014 |
| BOOKMAKING, HORSE & SPORT, ARRESTED, ADULT, 2014 |
| BOOKMAKING, HORSE & SPORT, ARRESTED, JUVENILES, 2014 |
| Colon/Rectal Srg, Other, 2016 |
| COVERAGE INDICATOR, 2014 |
| COVERAGE INDICATOR, ALL, 2014 |
| CURFEW, LOITERING VIOLATIONS, JUVENILE, ARRESTED, ALL, 2014 |
| DO's, Inactive, < 35, 2016 |
| Durable medical equipment reimbursements per enrollee: Price, age, sex & race-adjusted, 2016 |
| EMBEZZLEMENT, ARRESTED, JUVENILES, 2014 |
| Flag - the percentage of persons aged 65 and older is in the 90th percentile (1 = yes, 0= no), 2018 |
| Forensic Path, PC, Hosp FT Stf, 2016 |
| Gini Coefficient, 2014 |
| Hospitals per 100,000 people, Cardiac intensive care, 2016 |
| Households for which reports were received, as % of occupied units for Program 2=Public Housing, 2014 |
| Households for which reports were received, as % of occupied units for Program 2=Public Housing, 2015 |
| Households for which reports were received, as % of occupied units for Program 2=Public Housing, 2017 |
| Households for which reports were received, as % of occupied units for Program 2=Public Housing, 2016 |
| Households for which reports were received, as % of occupied units for Program 5=Project Based Section 8=202/PRAC, 2014 |
| Households for which reports were received, as % of occupied units for Program 5=Project Based Section 8=202/PRAC, 2016 |
| Households for which reports were received, as % of occupied units for Program 5=Project Based Section 8=202/PRAC, 2017 |
| Households for which reports were received, as % of occupied units for Program 5=Project Based Section 8=202/PRAC, 2018 |
| Households for which reports were received, as % of occupied units for Program 5=Project Based Section 8=202/PRAC, 2015 |
| Indicator of the presence of health-related drinking water violations. 0=No, 1=Yes |
| Industrial, self-supplied surface-water withdrawals, saline, in Mgal/d, 2015 |
| Industrial, self-supplied total withdrawals, total (fresh+saline), in Mgal/d, 2015 |
| Injury deaths, 2014 |
| Inptn Day in LT Chronic Disease, 2016 |
| Inptn Day in LT Intel Disablity, 2016 |
| Irrigation-Golf, acres irrigated, surface (flood), in thousand acres, 2015 |
| Licensed Beds, Long Term Hosp, 2016 |
| Livestock, surface-water withdrawals, fresh, in Mgal/d, 2015 |
| MD's, Fam Med Subsp,Other, 2016 |
| MD's, Family Prac Subsp, 75 +, 2016 |
| MD's, Gen Pract, Teaching, 2015 |
| Neurolgcal Surg, Other, 2016 |
| Nonspecialized-dependent county indicator, 2015 |
| Number of extreme heat days: Maximum Temperature, 105°F, 2014 |
| NUMBERS & LOTTERY, ARRESTED, JUVENILES, 2014 |
| Nursing Home Pers Tot,Vacancies, 2016 |
| Ob-Gyn Subspecs, Oth Prof Act, 2016 |
| Occupied units as the % of units available for Program 1=Summary of All HUD Programs, 2016 |
| OFFENSES AGAINST FAMILY AND CHILD, ARRESTED, JUVENILES, 2014 |
| Other Transmission Route Cases, 2018 |
| Percent of hospital beds within flood hazard area, 2016 |
| Percent of land covered by water, 2016 |
| Percentage of driving deaths with alcohol involvement. |
| Percentage of households in which the older of the household head or spouse is 51 to 61 years of age for Program 5=Project Based Section 8=202/PRAC, 2015 |
| Percentage of households in which the race of the head of household is a combination of White, Black, Native American, Asian or Pacific Islander races for Program 2=Public Housing, 2014 |
| Percentage of households in which the race of the head of household is a combination of White, Black, Native American, Asian or Pacific Islander races for Program 2=Public Housing, 2015 |
| Percentage of households in which the race of the head of household is a combination of White, Black, Native American, Asian or Pacific Islander races for Program 2=Public Housing, 2016 |
| Public Health, PC, Hosp Residnt, 2016 |
| Public Supply, surface-water withdrawals, saline, in Mgal/d, 2015 |
| Public Supply, total withdrawals, saline, in Mgal/d, 2015 |
| Rate of delinquency cases per 1,000 juveniles |
| Summer Food Service Program participants (% pop), 2014 |
| SUSPICION, ARRESTED, ADULT, 2014 |
| SUSPICION, ARRESTED, ALL, 2014 |
| SUSPICION, ARRESTED, JUVENILES, 2014 |
| Transplant Surg, Administration, 2016 |
| Transplantation Surgery, 75 +, 2016 |
| VAGRANCY, ARRESTED, JUVENILES, 2014 |
| Vascular Med, Research, 2016 |
| Vascular Medicine, 35-44, 2016 |

| Average annual percent of diabetic Medicare enrollees age 65-75 having blood lipids (LDL-C) test: White: Rate, 2014 |
| --- |
| Average annual percent of diabetic Medicare enrollees age 65-75 having blood lipids (LDL-C) test: Overall: Rate, 2015 |
| Average annual percent of diabetic Medicare enrollees age 65-75 having blood lipids (LDL-C) test: White: Rate, 2015 |
| Average annual percent of Medicare enrollees having at least one ambulatory visit to a primary care clinician : Overall: Rate, 2014 |
| Domestic, publicly supplied per capita use, in gallons/day [DO-PSDel/PS-TOPop], 2015 |
| Percentile Percentage of persons below poverty estimate, 2018 |
| 4-year Hispanic pregnancy-related mortality ratio per 100,000 live births where at least 10 deaths (2015-2018) |
| Irrigation, acres irrigated, sprinkler, in thousand acres, 2015 |
| Outpatient facility reimbursements per enrollee : Age, sex & race-adjusted, 2015 |
| Occupied units as the % of units available for Program 5=Project Based Section 8=202/PRAC, 2017 |
| Occupied units as the % of units available for Program 5=Project Based Section 8=202/PRAC, 2018 |
| Percent having an ambulatory visit within 14 days of discharge to home: Rate : medical cohort, 2015 |

Figure S1. Example of a Bayesian network graph of the relationships among total population PRM ratio (PRM1518, highlighted in orange) and its top correlates from all PHE domains. The arcs directed to this variable originates from factors that contribute to a healthy food environment (v133_rawvalue).


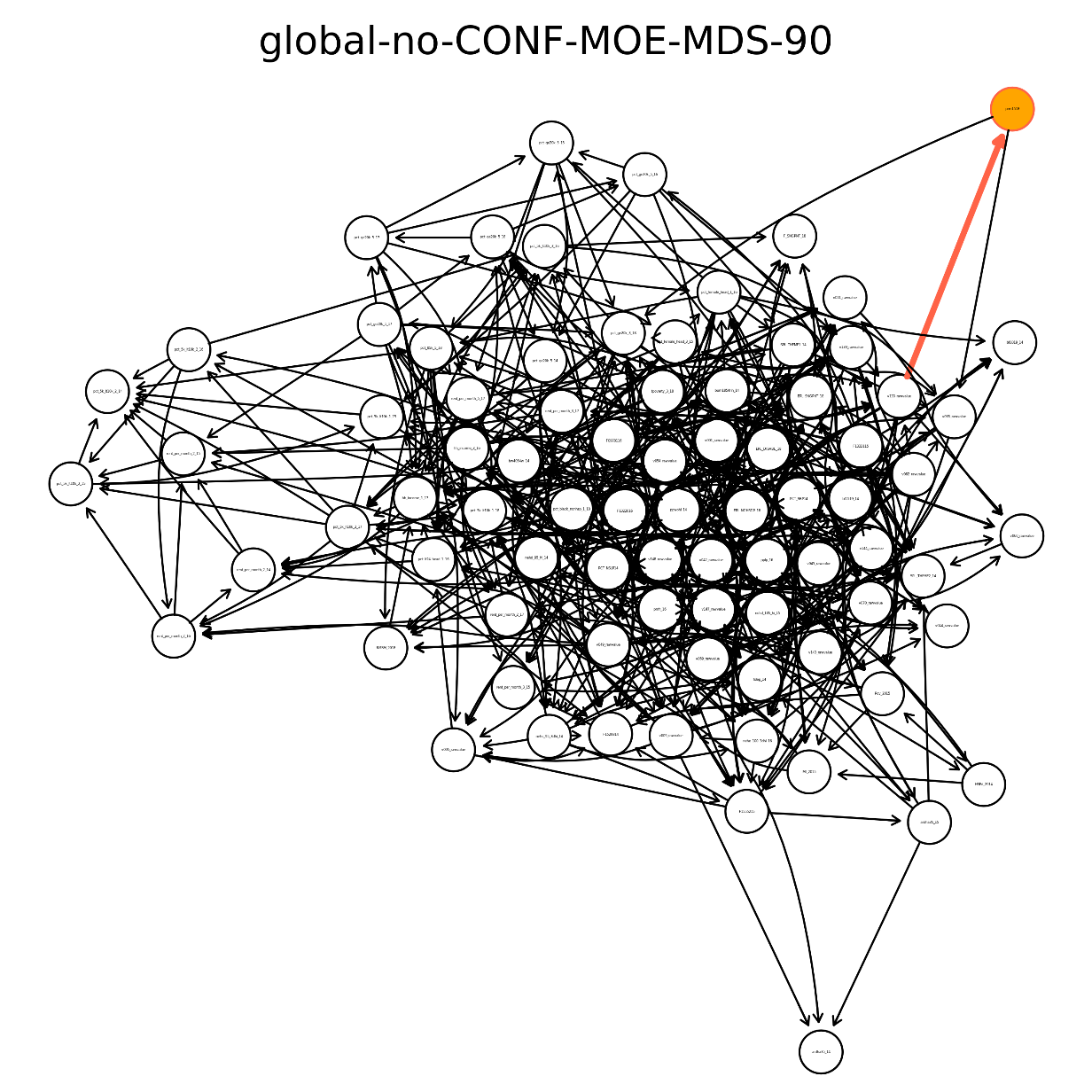

Supplement: Supplementary file 1 — Additional file 1. [file 12889_2022_14397_MOESM1_ESM.docx]
